# Strain-resolved biology of *Lactobacillus johnsonii* across host systems

**DOI:** 10.3389/fvets.2026.1868892

**Published:** 2026-08-10

**Authors:** Minghui Li, Jinling He, Jianmin Chai, Feilong Deng, Ying Li

**Affiliations:** Guangdong Provincial Key Laboratory of Animal Molecular Design and Precise Breeding, School of Animal Science and Technology, Foshan University, Foshan, China

**Keywords:** extracellular vesicles, host adaptation, intestinal barrier, *Lactobacillus johnsonii*, postbiotics, strain specificity, synthetic microbiome, weaned piglets

## Abstract

*Lactobacillus johnsonii* is widely described as a probiotic species, but current evidence supports a strain-resolved view. Its biological effects depend on strain background, host niche, diet, challenge context and delivery format, including live cells, cell-derived structures and postbiotic fractions. This review synthesizes comparative genomic, mechanistic and intervention studies on *L. johnsonii*, using weaned piglets as the main translational reference because barrier injury, inflammation, microbial disruption, diarrhea and impaired growth often coincide after weaning. Piglet studies show that selected strains can reduce diarrhea or pathogen burden, improve barrier-related markers and alter immune, oxidative and metabolic responses. Cross-host work links strain variation to surface architecture, adhesion, exopolysaccharides, redox activity, extracellular vesicles and other non-cellular effectors. These mechanisms recur around colonization resistance, epithelial barrier support, immune regulation, microbial metabolite production and control of cellular stress. The strongest claims connect a named strain with a defined host context and a measurable phenotype. Evidence is weaker when species-level abundance, multistrain formulations or rodent disease models are used to infer direct efficacy in livestock. This review frames *L. johnsonii* as a host-associated, strain-dependent bacterial lineage rather than a uniform probiotic entity. This review is primarily focused on piglet-centered veterinary applications; therefore, cross-host studies are discussed mainly as mechanistic support rather than as direct evidence of efficacy in piglets or other livestock species.

## Introduction

**Figure 1:**
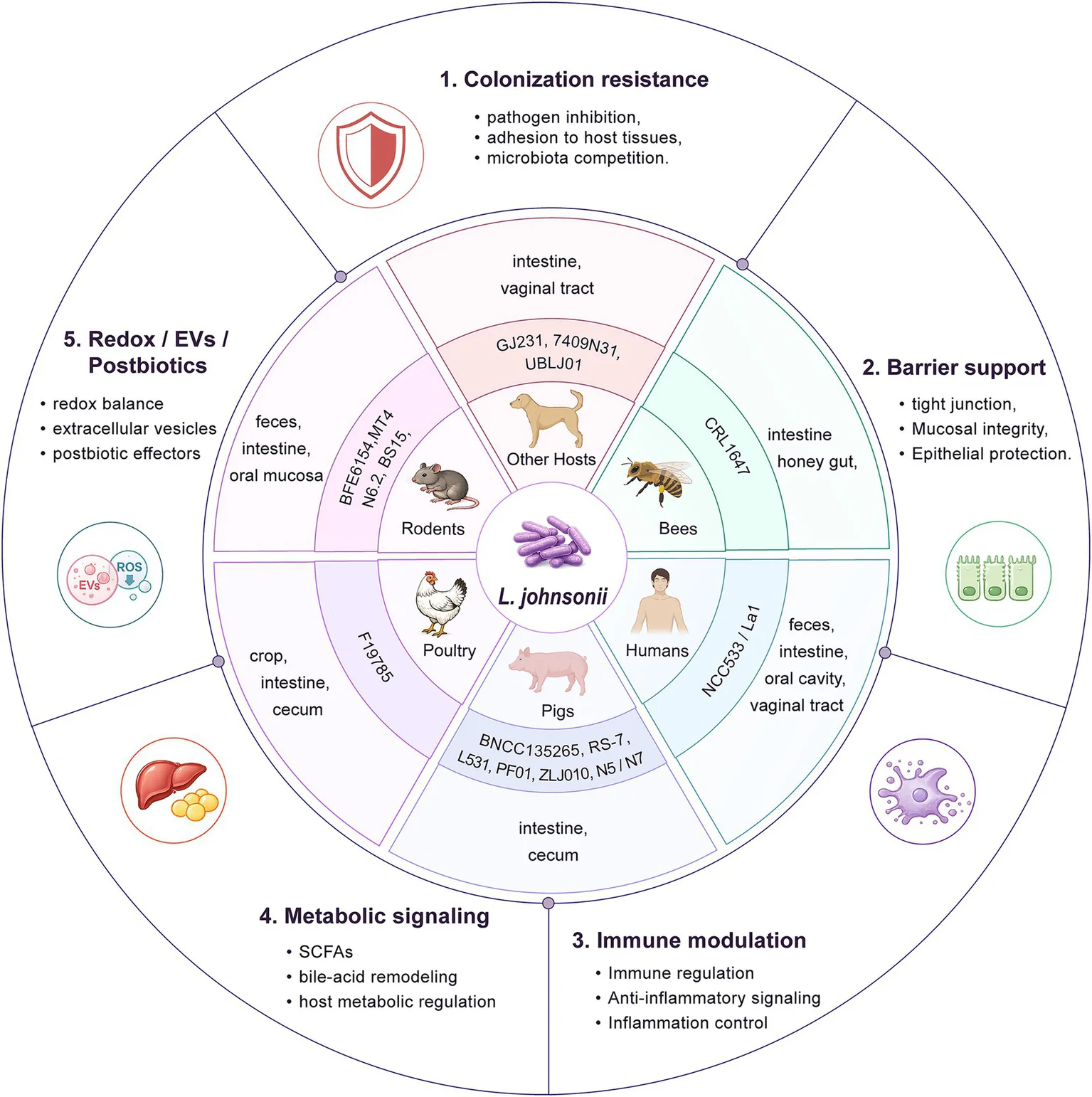
Species-strain-host-function overview of *L. johnsonii*. The figure summarizes major host-associated niches, representative strains and principal functional axes supported by the literature. *L. johnsonii* is shown as a host-associated species with strain-dependent functional outputs, including colonization resistance, barrier support, immune remodeling, metabolic signaling, and redox, extracellular-vesicle and postbiotic biology.

*Lactobacillus johnsonii* is often placed under the probiotic label, but that label can hide the strain-level biology that determines host responses. Hereafter, *L. johnsonii* is used for the species. The organism has been recovered from multiple host systems, including humans, pigs, poultry, rodents, dogs and bees, as well as from host-associated niches such as the intestine, oral mucosa and vaginal tract. Studies have linked *L. johnsonii* to infection control, inflammation, metabolic disturbance, neuroimmune signaling and mucosal injury. These observations do not make the species a single functional unit. At the species level, *L. johnsonii* recurs in host niches where epithelial surfaces face dietary, microbial or inflammatory pressure. At the strain level, activity depends on genome content, surface architecture, oxygen handling, metabolic capacity and the host challenge under study. The species-strain-host-function organization used in this review is summarized in Figure 1.

This distinction becomes especially important in weaned piglets. Post-weaning diarrhea develops during a short period in which diet change, social and environmental stress, immature digestion, pathogen exposure and microbiota disruption meet epithelial barrier failure, inflammatory activation and oxidative pressure. Classical piglet studies have described cytokine induction after weaning, increased permeability, lower tight-junction protein expression, epithelial stress signaling and immune-enriched transcriptional programs (1–4). Weaning also reshapes bacterial composition and fermentation-related metabolites (5, 6). A candidate *L. johnsonii* strain for this setting should therefore be evaluated as more than an acid- and bile-tolerant isolate. Its relevance depends on whether it can operate in the post-weaning gut and modify linked barrier, immune, microbial and metabolic disturbances without worsening inflammation or digestion.

Direct piglet evidence is still concentrated in a small number of named strains. BNCC135265, RS-7 and L531 provide the clearest strain-resolved examples, although their endpoints and experimental settings differ. BNCC135265 was associated with reduced post-weaning diarrhea and higher colonic tight-junction expression through an endocannabinoid-linked mechanism (7). RS-7 affected growth-related, antioxidant and immunoglobulin readouts, whereas L531 reduced *Salmonella* burden and intestinal injury in infection models, with changes in short-chain fatty acid profiles, mucosal IgA, inflammatory signaling, autophagy and mitochondrial injury pathways (8, 9). These studies support the practical relevance of *L. johnsonii* in pig production. They also show why efficacy cannot be inferred from the species name alone.

Cross-host work helps explain the piglet signal without replacing it. Rodent colitis models link *L. johnsonii* to macrophage and T-cell regulation, innate immune signaling, antioxidant responses, epithelial stress pathways and microbial metabolites (10–13). Studies using extracellular vesicles, nanovesicles, phospholipids, heat-killed cells and other postbiotic preparations show that some effects may be mediated by bacterial structures or products rather than by viable colonization alone (14–16). These papers are useful as mechanistic bridges. They should not be read as evidence that every *L. johnsonii* strain will produce the same phenotype in pigs, poultry or humans.

The review uses weaned piglets as the main translational reference and treats cross-host studies as mechanistic context. The central question is where a named *L. johnsonii* strain can be linked to a defined host setting and a measurable phenotype. Studies that meet this standard receive greater weight. Species-level abundance shifts, undefined multistrain preparations and distant disease models are interpreted more cautiously. From a synthetic microbiome perspective, strain-resolved *L. johnsonii* evidence can help identify candidate functional modules for rational consortium design, including colonization resistance, epithelial barrier support, immune remodeling, metabolic signaling, redox regulation, and extracellular-vesicle or postbiotic delivery. Rather than treating *L. johnsonii* as a uniform probiotic species, these modules can guide the selection of defined strains or strain-derived effectors that match specific veterinary targets. This approach may support the development of more precise microbial interventions for piglet gut health, while still requiring direct validation in the target livestock host.

### Nomenclature, host ecology and the argument for strain resolution

The 2020 reorganization of the genus *Lactobacillus* changed the names of many familiar lactobacilli, but *Lactobacillus johnsonii* remained within the amended genus *Lactobacillus*. This review therefore uses *Lactobacillus johnsonii*, abbreviated as *L. johnsonii*, because it remains the accepted species name (17). The broader taxonomic revision still matters because historical *lactobacillus* groupings can mask phylogenetic and ecological heterogeneity. In *L. johnsonii*, that heterogeneity is most evident at the host and strain levels. Comparative analyses of isolates from birds, humans, rodents and other hosts have shown host-associated clustering, suggesting that the species is shaped by host environments rather than by a generic lactic-acid bacterial niche (18). More recent genome and transcriptome work across vertebrate isolates extends this view, highlighting host-associated differences in surface proteins, secretion-related systems, tyramine-related modules and traits linked to adhesion, acid tolerance and nutrient use (19).

The current evidence base also reveals host association through the strains that recur across the literature. The human intestinal strain NCC533, also known as La1 in several studies, became a reference for genome interpretation, surface biology and early clinical testing (20–22). The poultry strain FI9785 was studied as a defined competitive-exclusion organism against bacterial pathogens, with effects that differed by pathogen species (23). Pig-associated strains include PF01, ZLJ010, L531, RS-7 and N5/N7, which appear in genome reports, piglet intervention studies or rodent bridging models (7, 8, 24–26). N6.2, isolated in the context of diabetes-resistant rats, has generated a separate literature around redox biology, extracellular vesicles, phospholipids and beta-cell or immune phenotypes (15, 27, 28). Oral MT4 and bee-derived CRL1647 extend the ecological map beyond the gut (29, 30). These strains should not be treated as interchangeable evidence for one species-level behavior. The representative strains, host contexts and evidence anchors used throughout this review are summarized in Table 1.

**Table 1:** Representative *L. johnsonii* strains and evidence anchors.

Strain or preparation	Source or main host context	Main evidence anchor	Interpretive value
BNCC135265	Weaned piglets	Reduced post-weaning diarrhea index, increased colonic ZO-1, occludin and claudin-1, DAGLB/endocannabinoid signal	Direct piglet barrier and diarrhea evidence
RS-7	Pig source, weaned piglets and DSS mice	Improved growth, immunoglobulins and antioxidant defenses in piglets. TLR4/MyD88/NF-κB and Nrf2-related effects in mice	Piglet performance plus mechanistic bridge
L531	Pig source, Salmonella-challenged piglets	Reduced Salmonella burden, fever and diarrhea frequency. Preserved SCFAs and affected inflammasomes, NF-κB, ER stress, autophagy and mitophagy	Best pathogen-challenge piglet series
PF01	Piglet feces	Genome resource, adhesion and safety-oriented comparative genomics	Pig-associated candidate requiring direct efficacy work
ZLJ010	Healthy sow feces	Complete genome and comparative genomic characterization	Resource for pig-source genomic adaptation
N5/N7	Heat-stress resistant pigs	WGS, stress tolerance, EPS and gut barrier/immune effects in rodent models	Host-source bridge between pig origin and mechanistic testing
FI9785	Poultry	Defined competitive exclusion against selected poultry pathogens. EPS affects adhesion and stress resistance	Classic example of pathogen-specific colonization resistance
N6.2	Diabetes-resistant rat lineage	H2O2/redox, extracellular vesicles, phospholipids, β-cell and immune signaling	Mechanistic platform for non-cellular effectors
BFE6154	Fermented milk	Diet-induced hypercholesterolemia and cholesterol transport modulation	Metabolic evidence outside pig systems
MT4	Murine oral mucosa	Chitinase activity and protection against oral Candida-associated mucosal injury	Emerging non-gut mucosal niche

Genomic data explain part of this heterogeneity. The *L. johnsonii* pangenome contains conserved regions required for core physiology and variable regions encoding host-adaptive traits, transport systems, carbohydrate use, surface structures, CRISPR arrays and prophage-associated modules. In ZLJ010, genome analysis emphasized an open pangenome, transport and defense repertoires, prophage content and CRISPR-related loci, supporting its interpretation as a pig-associated probiotic candidate (25). PF01, a piglet feces isolate, carried features linked to adhesion, bile adaptation and inhibition of enteric pathogens, and was later included in comparative safety analysis (24, 31). N5 and N7 from swine were characterized by acid and bile tolerance, inhibitory capacity, exopolysaccharide production, carbohydrate-active enzyme genes and stress-related gene content (26). Poultry genome studies have connected host-matched strains with early performance and colonization traits, and have highlighted core SNPs, integrative elements and CRISPR-linked variation as part of host adaptation (32). These genomic features define a pool of possible adaptations and effectors, but their biological meaning depends on strain-level phenotyping in the relevant host context.

Mobile elements and phage-defense systems influence strain interpretation because they affect genome stability, functional consistency and biosafety assessment. A study of host-specific diversity reported a large chromosomal inversion and phage-resistance mechanisms among *L. johnsonii* strains, linking genome architecture with host origin and viral pressure (33). Genome mining of CRISPR-Cas systems further revealed diverse targeting histories across *L. johnsonii* genomes, consistent with differences in exposure to phages, plasmids and other invading genetic elements (34). These features are often absent from efficacy narratives, yet they are relevant to strain comparability. Instability in a surface structure, phage-defense region or plasmid-linked trait during scale-up could make the administered organism differ from the strain characterized in a small laboratory experiment.

Nutrient adaptation provides another route from genome to phenotype. The NCC533 genome suggested a host-dependent lifestyle, with transporters, peptidases, bile salt hydrolase potential and surface proteins suited to residence in the upper gastrointestinal tract (20). L-alanine auxotrophy in NCC533 showed that host adaptation can also create nutritional dependence, so adaptation and limitation may be encoded in the same genome (35). Other strains have been selected or interpreted through bile salt hydrolase activity, carbohydrate utilization or metabolite production. CCFM1376 improved hypercholesterolemia through high bile salt hydrolase activity and bile acid remodeling in mice (36). The Hanwoo-derived strain 7,409 N31 carries carbohydrate-utilization potential relevant to feed-additive development (37). These findings should not be treated as direct evidence of piglet efficacy. They identify traits that need to be measured when a strain is transferred across host species, diet types or production systems.

Strain documentation is the minimum condition for fair evidence synthesis. Studies are easier to interpret when they identify the strain, document its source, describe verification methods, report viability or preparation format, and avoid treating species-level abundance as proof of causality. Several studies meet this standard by using named strains, genome-level resources or direct intervention designs. Others report an increase in *L. johnsonii* after a dietary or plant-derived intervention and then infer that it contributes to the observed phenotype. Such studies can generate useful hypotheses, especially when followed by antibiotic depletion, monoassociation, metabolite rescue or strain recovery. Without those steps, they should be considered associative rather than causal (38). Strain resolution is therefore not merely a technical refinement. It is the basis for comparing studies and for moving from genomic potential to host-level function (39).

## Molecular effectors linking strain background to host response

### Exopolysaccharide and surface architecture

The first host-facing feature of *L. johnsonii* is its cell surface. Before a strain acts through metabolite production or broader microbiota remodeling, it encounters mucus, epithelial cells and neighboring microbes through exopolysaccharides, S-layer-associated structures, adhesins and surface-exposed or released proteins. FI9785 shows how much this surface layer can vary in function. Mutational work found that its exopolysaccharide layer altered surface charge, hydrophobicity, aggregation, biofilm formation, adhesion and stress resistance (40). EPS is sometimes treated as a simple adhesion determinant, but this study points to a more nuanced interpretation. Surface polysaccharides can impose trade-offs. A structure that improves stress tolerance may also change host binding, aggregation or biofilm behavior, and the balance is likely to differ among strains.

Surface and released proteins add another layer to this interaction. Work on La1/NCC533 identified GroEL as a cell-surface-associated protein with pH-dependent binding to mucus and epithelial cells, interaction with CD14-linked immune recognition, and aggregation with *Helicobacter pylori* (41). More recent work with *L. johnsonii* MG showed that released GAPDH binds the tight-junction protein JAM-2 and upregulates barrier-associated genes, with defined peptide regions involved in binding (42). Subsequent receptor-focused work supported the view that surface-exposed or released proteins from *L. johnsonii* can act directly on epithelial junction biology (43). These findings help explain why live cells, culture supernatants, extracellular vesicles and heat-killed fractions sometimes produce overlapping effects. In such cases, the active signal may be a bacterial structure or protein that remains functional outside a replicating cell.

Taxonomy alone cannot predict adhesion, persistence or pathogen competition. PF01 was reported to show proximal small-intestinal adhesion and pathogen-inhibitory traits in genome-linked work (24). Swine strains N5 and N7 showed acid and bile tolerance together with candidate EPS- and carbohydrate-related traits (26). Beagle-derived GJ231 and vaginal UBLJ01 displayed distinct tolerance, adhesion, aggregation, biofilm or antimicrobial profiles in their respective niche-oriented assays (44, 45). These assays are useful for early strain screening because they indicate whether a strain may persist, contact the epithelium or compete with pathogens. They do not establish whether the host response will be barrier repair, inflammatory activation, immune tolerance or no measurable phenotype. That question requires a defined host context and an intervention design.

### Redox modules and organelle stress

The N6.2 studies provide a useful entry point for redox biology in *L. johnsonii*. They showed that hydrogen peroxide production depends on strain background, flavin reductase components and oxygen availability, rather than on the species label alone (28). This matters because the same redox capacity may shape host oxidative signaling and indoleamine 2,3-dioxygenase-related pathways in different ways across models. In epithelial and inflammatory settings, several strains have been associated with lower oxidative-injury readouts. RS-7 in DSS-treated mice was linked to microbial metabolite shifts, a lower abundance of the lipid annotated as LPS18:0, higher DL-lysine, Keap1 suppression and activation of the Nrf2/NQO1/HO-1 axis (12). Extracellular vesicles derived from *L. johnsonii* also activated Nrf2/HO-1 in epithelial cells and alleviated colitis in mice (46). These findings do not justify describing *L. johnsonii* simply as antioxidant. They show that redox output depends on oxygen tension, bacterial metabolism, host cell type and inflammatory state. A strain may produce oxidants in one setting and limit oxidative injury in another.

When microbial challenge damages the epithelium, redox biology becomes linked with intracellular stress control. L531 studies in *Salmonella* models make this connection explicit. One study reported that L531 suppressed NOD1/2-RIP2 activation, relieved ER stress and restored autophagy-related degradation during intestinal injury (47). Another found reduced enteritis with lower NLRC4 and NLRP3 inflammasome activity, less NF-κB activation and altered SQSTM1-dependent mitophagy, together with improved handling of damaged mitochondria (48). A related diarrhea model associated L531 with reduced iron overload and ROS, as well as restored Nrf2/HO-1 signaling (49). These findings place *L. johnsonii* effects within epithelial stress programs, rather than only in extracellular pathogen competition or cytokine suppression.

A similar stress-control logic appears in more distant models, although those data should be treated as mechanistic rather than translational evidence for piglet diarrhea. BS15 studies connected barrier improvement with reduced LPS exposure, lower hepatic inflammation, mitochondrial protection and memory-related neurochemical endpoints (50, 51). JNU3402 studies linked *L. johnsonii* with hepatic lipid and aging phenotypes through PKA-SREBP1c and PGC-1α-PPARα-SIRT1-p53 signaling (52, 53). These systems do not model post-weaning intestinal disease directly. Their value lies in showing how changes at the gut interface may propagate to liver, brain or aging-related physiology when microbial metabolism and barrier function shift. For livestock questions, they are best used to nominate pathways for testing, not to infer efficacy.

### Extracellular vesicles, phospholipids and postbiotics

The N6.2 literature also shows how non-cellular effectors can carry strain-specific signals. N6.2-derived nanovesicles reduced apoptosis in human *β*-cells and promoted AHR-linked and IL-10-associated immune signaling in cell models (14). Bile exposure increased N6.2 extracellular vesicle production while preserving immunomodulatory properties, connecting gut chemical cues to vesicle output (54). Proteomic and functional studies identified EV-associated domains such as Sdp-SH3b2 with antiviral activity against murine norovirus, including OAS pathway activation and IL-10-like tolerance responses (54). Biomarker studies of N6.2 nanovesicles identified proteins involved in systemic distribution and immune recognition (55). These papers make the cell-free fraction part of *L. johnsonii* biology, not a by-product.

The same theme appears in colitis and piglet-associated work. Dietary *L. johnsonii* EVs improved acute colitis in mice by entering epithelial cells, activating Nrf2/HO-1, preserving barrier function and reshaping the microbiota (46). Piglet-associated work reported that extracellular vesicles from *L. johnsonii* promoted barrier homeostasis by enhancing M2 macrophage polarization, inhibiting ERK signaling in macrophages and limiting epithelial NLRP3 activation, with improved ZO-1 and occludin expression (56). Another study of *L. johnsonii*-derived EVs carrying GAPDH linked macrophage MAPK/STAT3 inhibition, M2 polarization, macrophage EV signaling through TLR4 and epithelial protection in ulcerative colitis models (57). Taurine-linked EV effects added Th17/Treg balance and IgA/IgG coating as mucosal immune endpoints (58). The repeated pattern is macrophage-epithelial dialogue driven by packaged proteins, lipids or metabolites.

Postbiotic and lipid fractions widen the same argument. N6.2 phospholipids induced immature or tolerogenic dendritic-cell states, altered migratory programs and promoted T-cell anergy after cognate interaction (59). In alcohol-associated liver disease models, heat-killed *L. johnsonii* activated gut innate immunity through a NOD2-RIP2, IL-23 and IL-22 axis, restored Paneth and goblet cell functions, improved barrier markers, corrected microbial imbalance and reduced liver injury (16). This matters for translation because live bacteria face problems of stability, colonization unpredictability, antibiotic co-use, phage exposure and safety concerns in compromised hosts. Postbiotics and EVs may avoid some of these barriers, but they introduce questions about dose standardization, cargo variability, manufacturing and off-target immune effects.

### Small molecules and microbial metabolism

Metabolite-centered studies have moved *L. johnsonii* research beyond taxonomic association. SCFAs remain the most familiar entry point, but the useful information lies in which acid changes, where it acts and which receptor or tissue compartment is involved. In piglets challenged with *Salmonella*, L531 helped preserve intestinal SCFA profiles (8). In DSS colitis, GLJ001 increased SCFA-producing taxa and raised acetate, propionate and butyrate, with reduced M1 macrophage polarization through GPR41/43-related signaling (60). A culture supernatant from *L. johnsonii* produced a related effect by increasing propionate-associated signals and suppressing p38-MAPK-dependent M1 polarization (61). In hyperuricemic mice, a mouse-derived *L. johnsonii* strain increased butyrate, suppressed Wnt5a/b/*β*-catenin signaling, upregulated intestinal ABCG2 and enhanced urate excretion (62). These studies explain why total SCFA concentration is a limited endpoint. The same metabolite class can support different outcomes depending on disease setting, receptor engagement and tissue location.

Other metabolic routes extend this logic. High-BSH CCFM1376 improved hypercholesterolemia in mice by increasing unconjugated bile acids and fecal bile acid excretion, suppressing ileal FXR-FGF15 signaling and upregulating hepatic CYP7A1 (36). In high-fat diet-associated colorectal cancer models, *L. johnsonii* BSH activity increased chenodeoxycholic acid and was linked to tumor mitochondrial dysfunction, increased ROS and apoptosis (63). In osteoarthritis models, mulberry water extract increased *L. johnsonii* and hyodeoxycholic acid, with macrophage rebalancing and joint protection (64). Tryptophan and peptide metabolism add another layer. In *β*-glucan-fed colitis models, *Bacteroides uniformis* supported *L. johnsonii*-dependent indole-3-lactic acid production, which activated AhR and IL-22 signaling (38). In chronic kidney disease models, loss of *L. johnsonii* and indole-3-aldehyde was associated with inflammatory and fibrotic injury, and supplementation with either *L. johnsonii* or indole-3-aldehyde reversed part of this phenotype (65). In chronic kidney disease with secondary hyperparathyroidism, *L. johnsonii* produced cyclo(Pro-Trp), activated CaSR/TRPV4-related pathways and promoted intestinal calcium absorption, an effect not reproduced by *L. reuteri* or *L. murinus* (66). Pan-cancer immunotherapy work identified cooperation between *L. johnsonii* and *Clostridium sporogenes* in indole-3-propionic acid production, with effects on Tcf7 enhancer acetylation and progenitor exhausted CD8 T-cell programs (67). The common point is not that *L. johnsonii* carries a single metabolic signature. Named strains can participate in substrate- and community-dependent metabolic networks.

Partial or negative findings belong in the same interpretation. In a piglet PEDV study, *L. lactis* and acetate protected the intestinal barrier through GPR43 and PI3K/AKT-related immune and epithelial effects, whereas the *L. johnsonii* group did not reproduce the same protection (68). More broadly, PEDV-focused veterinary literature has framed natural bioactive compounds partly through oxidative-stress pathways, suggesting that redox injury should be treated as a disease-context endpoint when microbial, postbiotic or metabolite-based interventions are evaluated in PEDV-challenged piglets (69). In a postoperative Crohn disease trial, *L. johnsonii* LA1 was well tolerated but did not significantly reduce early endoscopic recurrence, clinical recurrence, histology scores or CRP (22). These studies make the literature more credible rather than weaker. They show that the presence of *L. johnsonii*, or even plausible metabolic capacity, is not enough to predict efficacy. The active molecule, strain background, disease stage, host immune state, route of delivery and neighboring microbiota determine whether the outcome is protective, neutral or ineffective.

## Functional axes across host systems

### Colonization resistance and pathogen control

L531 gives the clearest piglet example of colonization resistance. In *Salmonella Infantis* challenge, it lowered fecal and intestinal *Salmonella* burden, reduced splenic translocation and improved growth-related outcomes (8). The companion mechanistic studies suggest that this effect is not explained only by direct bacterial antagonism. Mucosal sIgA, inflammasome suppression, NF-κB control, ER stress, autophagy, mitochondrial injury and iron-redox signaling all appear in the L531 series (47–49). Pathogen load and host tissue resilience should therefore be read together. A lower pathogen burden reduces injury, but a less inflamed, less leaky and metabolically steadier epithelium also makes the niche harder for pathogens to exploit.

Poultry studies strengthen this theme and show its limits. FI9785 reduced *Clostridium perfringens* colonization and persistence more clearly than it reduced *Salmonella Enteritidis*, with partial effects against *E. coli* O78:K80 (23). A broiler *Salmonella Sofia* study reported lower *Salmonella* and *C. perfringens* after early *L. johnsonii* administration, with increased *Lactobacillus* by day 35 and altered volatile fatty acids (70). A more recent *Ascaridia galli* chicken study reported early improvements in feed intake, gain and feed conversion, lower worm burden, reduced FITC-dextran permeability, increased ZO-2 and occludin, lower IL-6, IFN-*γ* and caspase signals, and a waning effect when *L. johnsonii* shedding declined (71). Persistence, timing and target pathogen therefore matter as much as nominal strain identity.

Pathogen control is not confined to the intestine of mammals or birds. Oral MT4 reduced *Candida*-associated lesions, protected E-cadherin distribution and keratinized layer integrity, damaged fungal cell-wall architecture through chitinase activity, and suppressed *Candida*-induced enterococcal expansion in the oral mucosa (29). Earlier MT4 work showed inhibition of *Candida* growth, biofilm formation and yeast-to-hypha transition through pH-dependent and pH-independent soluble factors (72). In bees, CRL1647 feeding improved brood area, colony numbers and honey storage, likely reflecting host-adapted fermentation or microbial support in a non-mammalian gut (30). These examples broaden the species map. They should not be used in piglet efficacy claims unless a comparable feature, such as niche competition, secreted antimicrobial activity or stable mucosal persistence, is tested in pigs.

### Barrier support and epithelial repair

Barrier support is the functional axis that most directly connects *L. johnsonii* to post-weaning diarrhea. In the BNCC135265 piglet study, a lower diarrhea index coincided with higher colonic ZO-1, occludin and claudin-1, and with endocannabinoid-system remodeling through DAGLB (7). The study is important because it joins a clinical endpoint to barrier proteins and a candidate host signaling pathway. It also defines the boundary of current evidence. Inflammation markers were not the main driver, and long-term production outcomes were limited. The result supports a barrier-focused mechanism, not a universal anti-inflammatory label.

Piglet background studies explain why tight junctions are meaningful endpoints. Early weaning lowers occludin, claudin-1 and ZO-1 expression, increases permeability and activates inflammatory and MAPK pathways (2). DAO markers and TGF-β-related repair signals change during the same period (3). Feed intake, gut architecture, microbial maturity and permeability shift over time after weaning, so intervention timing can alter what is measurable (73, 74). A *L. johnsonii* study that measures diarrhea without tight junctions misses mechanism. A study that measures tight junctions without diarrhea or growth misses field relevance. Strong piglet studies need both.

Rodent and cellular studies offer barrier mechanisms that can be tested in piglets. RS-7 in DSS mice improved goblet cells, MUC2, ZO-1 and occludin, with lower TLR4/MyD88/ NF-κB activity (11). GLJ001 restored ZO-1 and occludin and reduced M1 macrophage polarization through SCFA-related signaling (60). N5 improved mucosal immunity and barrier integrity in DSS models and was linked to HSP70, Peyer’s patch dendritic cells and Treg/Th17 balance (75). *L. johnsonii* MG-GAPDH bound JAM-2 and enhanced tight-junction gene programs (42). EVs from *L. johnsonii* entered epithelial cells and activated Nrf2/HO-1 to preserve barrier function (46). These mechanisms are credible bridges, especially when the effector is defined. They should still be tested against piglet endpoints before being used to design a product for post-weaning diarrhea.

### Immune remodeling

The immune evidence is more varied than the phrase immunomodulation suggests. In piglets, RS-7 increased serum total protein, albumin and immunoglobulins, and improved mucosal antioxidant status (9). L531 increased mucosal sIgA during *Salmonella* challenge and reduced inflammasome and NF-κB signaling in intestinal tissues (48). Porcine monocyte-derived dendritic cell work showed that *L. johnsonii* upregulated TLR2/TLR6, promoted dendritic-cell maturation and shaped CD4 T-cell responses, whereas culture supernatant favored Th2/Treg-related and IL-10-associated patterns (76). These pig-relevant data suggest that *L. johnsonii* can act on innate recognition, antigen-presenting cells, mucosal antibodies and inflammatory tone, but the direction depends on viable cells versus supernatant and on the inflammatory context.

Macrophages appear repeatedly in recent *L. johnsonii* studies, but the upstream signal differs by model. In DSS colitis and human ulcerative colitis-related work, *L. johnsonii* activated TLR1/2-STAT3 signaling and CD206 + IL-10-producing macrophages, reducing inflammation and supporting barrier repair (10). GLJ001 and culture supernatant studies connect SCFAs or propionate to reduced M1 polarization and lower inflammatory cytokines (60, 61). EV studies point to GAPDH-carrying vesicles, macrophage MAPK/STAT3 inhibition, M2 polarization and secondary macrophage EV signaling to epithelium (57). Piglet-derived *L. johnsonii* vesicle work also placed M2 polarization upstream of epithelial NLRP3 closure and tight-junction recovery (56). The same cell type appears across studies, but the signal can be a surface receptor, a metabolite, vesicle cargo or a heat-killed bacterial structure.

T-cell and mucosal antibody pathways are part of the same pattern. N5 studies linked *L. johnsonii* to splenic and mucosal Treg/Th17 balance during DSS colitis (75, 77). Taurine-linked EV work connected *L. johnsonii* extracellular vesicles to Th17/Treg rebalancing, PIGR and FcRn regulation, and abnormal IgA/IgG bacterial coating in IBD models (58). N6.2 phospholipids induced dendritic-cell states that drove T-cell anergy-like programs (15). Heat-killed *L. johnsonii* in alcohol-associated liver disease activated a NOD2-IL-23-IL-22 relay, increasing antimicrobial peptides and barrier repair (16). These studies suggest immune remodeling rather than simple suppression. In a vulnerable piglet, immune support may require improved antibody and epithelial defense without driving excessive Th17, inflammasome or neutrophil-mediated injury.

Neutrophil biology adds a newer layer. N5 reduced NETosis, bacterial translocation and gut-liver injury in DSS colitis, with DNase I and cell-free supernatant experiments helping separate NET-mediated damage from other inflammatory pathways (13). Post-weaning diarrhea models increasingly implicate inflammasomes, pyroptosis and neutrophil-rich inflammation in pathogen-driven injury, even in studies that do not involve *L. johnsonii* (78). L531 already links *Salmonella* protection to inflammasome and mitochondrial stress control (48). A future piglet study that measures neutrophils, NETs, pyroptosis and mitochondrial stress alongside diarrhea and pathogen burden would connect rodent-level mechanism with piglet-level translation.

### Metabolic signaling and cross-organ effects

The metabolic literature places *L. johnsonii* in disease areas beyond intestinal infection. BFE6154 lowered circulating and hepatic cholesterol, changed intestinal cholesterol transporter signals and increased fecal cholesterol excretion (79). CCFM1376 lowered cholesterol through BSH-driven bile acid deconjugation and FXR-FGF15-CYP7A1 remodeling (36). In high-fat diet-associated colorectal cancer models, *L. johnsonii* and BSH-mediated CDCA production reduced tumor burden through mitochondrial dysfunction and ROS-linked apoptosis (63). In parenteral nutrition-associated liver injury, *L. johnsonii* influenced BSH-linked bile acid circulation and hepatocyte apoptosis or fatty acid oxidation signals (80). These data define a bile-acid and lipid axis that may interact with diet composition in livestock, although direct piglet tests remain limited.

Tryptophan and indole metabolism provide a second axis. Beta-glucan cross-feeding work showed that *L. johnsonii* may require another microbe to release the substrate needed for indole-3-lactic acid production and AhR/IL-22 activation (38). CKD work with IAld and pan-cancer work with IPA show that indole-related metabolites can reach kidney or tumor-immune pathways when microbial partners and host disease states align (65, 67). Methamphetamine craving and schizophrenia-like behavior models also connect *L. johnsonii* to tyrosine or tryptophan-related metabolites and gut-brain signaling, although these are distant from the intestinal disease core (81, 82). The main lesson for gut biology is that *L. johnsonii* often works as part of a metabolic consortium. Feed ingredients, host diet, competing microbes and disease-associated substrates can decide whether a strain has access to the pathway under discussion (83).

Mineral and purine metabolism show strain selectivity in another way. In chronic kidney disease with secondary hyperparathyroidism, *L. johnsonii* generated cyclo(pro-trp) and promoted intestinal calcium absorption, whereas *L. reuteri* and *L. murinus* did not show the same effect (66). In hyperuricemia, *L. johnsonii* increased butyrate, upregulated ABCG2 and enhanced intestinal urate excretion, again with a near-neighbor comparison that did not fully reproduce the effect (62). These studies are not relevant to piglet diarrhea by disease category. They matter because they show how one *Lactobacillus* species can produce a metabolite or host response that close relatives do not. The same logic applies when *L. johnsonii* strains are compared in livestock.

Cross-organ models require restraint. BS15 studies link gut barrier and inflammation to liver steatosis and memory impairment (50, 51). EU03 connected gut microbiome remodeling, barrier restoration and SCFA or bile-acid metabolites to acute myocardial infarction outcomes (84). GKJ2 studies used maternal or intranasal exposure to reduce hyperoxia-induced lung injury in neonatal mice through gut-lung axis changes (85, 86). A neuroangiostrongyliasis model reported protection through microbiota-gut-brain mechanisms (87). Such work is important for microbiome research because it shows how mucosal bacteria may shape systemic physiology. However, cross-host mechanisms should be treated as testable hypotheses until they are directly evaluated in the target host, diet, delivery format and challenge context.

### Weaned piglets as the translational test case

Direct piglet evidence, piglet pathophysiology background and microbiome-intervention comparators should be separated. BNCC135265 reduced post-weaning diarrhea and increased colonic tight-junction expression through an endocannabinoid-linked pathway (7). RS-7 improved growth, feed conversion, immunoglobulins and intestinal antioxidant capacity, but did not directly report diarrhea rate, pathogen load or classical tight-junction markers (9). L531 lowered *Salmonella* burden and translocation, preserved SCFAs and improved growth under *Salmonella Infantis* challenge (8). A companion L531 study reduced diarrhea frequency and fever, increased mucosal sIgA and linked protection to inflammasome, NF-κB, mitochondrial and mitophagy pathways (48). These studies justify *L. johnsonii* as a candidate for post-weaning gut health. They do not yet define an optimized commercial protocol.

Piglet background studies supply the disease map. Weaning triggers early cytokine induction, villus and enzyme changes, altered permeability, lower tight-junction protein expression, DAO shifts, MAPK activation and immune-gene remodeling (1–4). The gut microbiome and metabolic profile shift during weaning, including fermentation and energy-related pathways (5). Feed intake can complicate interpretation because higher early intake may improve growth but raise diarrhea risk when digestive capacity lags behind nutrient load (74). Non-*L. johnsonii* interventions such as glutamine, lactate, sodium isobutyrate, iron route manipulation, binding proteins or *Bacillus*-based probiotics show that barrier repair, microbial metabolites, pathogen adhesion, inflammatory tone and epithelial renewal are tractable targets in this host (88–92). They are comparators, not evidence for *L. johnsonii*, but they help define endpoints that a strong *L. johnsonii* trial should measure. Recent piglet nutrition studies further broaden this comparator set: *Hermetia illucens* larvae full-fat meal combined with astaxanthin has been examined for large-intestinal microbiome and histomorphology outcomes, whereas chlorogenic acid supplementation in weaned piglets with intrauterine growth retardation has been linked to redox status, hepatic inflammation and mitochondrial function (93, 94).

A product-oriented piglet trial would need a narrower design than many exploratory studies. The strain should be identified by genome sequence, deposited or otherwise traceable, and screened for acquired antimicrobial resistance, virulence factors, plasmid mobility, hemolysis, biogenic amine production and transferable risk. Dose should be expressed as viable CFU at feeding and after storage, or as a chemically defined EV or postbiotic unit. The intervention window should distinguish prevention before weaning, support during the first week after weaning and treatment after diarrhea onset. Endpoints should include diarrhea scoring, fecal pathogen load, growth, feed intake, feed conversion, mortality or treatment use, intestinal histology, permeability, mucins, tight-junction proteins, sIgA, cytokines, inflammasomes or NETs, redox markers, mitochondrial or ER stress, and targeted metabolites such as SCFAs, bile acids and indoles. No single paper can measure everything, but without enough of these layers the field will keep producing studies that are difficult to compare.

Attribution is especially important in piglets. A Wuzhishan minipig DSS study found that a mixture of *L. crispatus*, *L. johnsonii* and *L. reuteri* alleviated colitis, improved ZO-1 and MUC1, increased TGF-beta1, lowered IL-1beta and oxidative markers, and reshaped microbial communities (95). The study is valuable because it uses a pig intestinal inflammation model. It cannot tell us which effects belong to *L. johnsonii*. The same caution applies to dietary interventions that enrich *L. johnsonii* but do not isolate and test it. These papers are useful for hypothesis generation. They become causal only when supported by monoassociation, add-back experiments, metabolite rescue, strain-specific depletion or a comparable design (Table 2).

**Table 2:** Evidence depth by host and functional module.

Host or model setting	Best-supported *L. johnsonii* modules	Main evidence level	Boundary for interpretation
Weaned piglets	Diarrhea reduction, barrier support, antioxidant and immune support, Salmonella load reduction, SCFA maintenance	Direct for a few named strains	Few strains and limited therapeutic designs
Pig infection and pig-cell models	NOD/NF-κB, inflammasomes, ER stress, mitophagy, dendritic-cell/T-cell interactions	Direct or close bridge	Often pretreatment and single challenge models
Poultry	Competitive exclusion, pathogen reduction, short-term barrier and parasite effects	Direct in poultry	Pathogen specificity and persistence vary
Rodent colitis	Barrier repair, macrophage polarization, Treg/Th17 balance, Nrf2, NETosis, ER stress	Mechanistic depth	Cannot stand in for piglet production efficacy
Rodent metabolic and cross-organ disease	Bile acids, indoles, SCFAs, calcium and urate transport, gut-brain/liver/kidney axes	Mechanistic and associative	Large distance from intestinal infection models
Human data	Clinical tolerance, IBD-related tissue or microbiome associations, limited RCT evidence	Mixed and context-dependent	LA1 Crohn trial was negative for recurrence prevention
EV/postbiotic systems	Macrophage-epithelial communication, Nrf2/HO-1, mucosal immunity, IL-22, phospholipid-induced tolerance	Rapidly growing mechanistic evidence	Standardized dose, cargo and safety remain unresolved

Durability remains a practical question. Chicken *A. galli* data show that *L. johnsonii* protection weakened when shedding declined by 30 days after infection (71). In pig production, this issue may be even more pronounced because feed transitions, antibiotics, zinc or acidifiers, pathogen pressure and environmental stress vary by farm. Live *L. johnsonii* may need synbiotic support, repeated dosing or diet-matched formulation. EVs and postbiotics may offer more stable delivery, but no piglet diarrhea study has yet matched the mechanistic sophistication of rodent EV work with production endpoints. The gap is not a reason to abandon the live strain approach. It is a reason to test live cells, killed cells, supernatant, EVs and purified effectors side by side in the same piglet model.

Safety in weaned piglets should not be assumed. *L. johnsonii* has a generally favorable profile in many experimental systems, and studies such as IDCC 9203 and PF01 safety analysis support the value of genome and phenotype screening (31, 96). Probiotic status remains context-dependent. *A. muciniphila* aggravated DSS-induced inflammation and epithelial disruption in weaned pigs, with worse growth, diarrhea, Th17-related inflammation, reduced SCFAs and impaired transport or tight-junction markers (97). That paper is not about *L. johnsonii*. It is a useful warning that a microbe with beneficial effects in one context can be harmful in another. For *L. johnsonii*, safety assessment should be strain-specific, host-specific and challenge-specific, especially in immunologically immature animals or during pathogen exposure.

### Cross-host evidence and limits of extrapolation

Rodent colitis models dominate the mechanistic literature because they allow controlled challenge, sampling and cellular validation. They have produced several coherent pathways. *L. johnsonii* can induce CD206 + IL-10 macrophages through TLR1/2-STAT3 signaling (10). RS-7 can suppress TLR4/MyD88/ NF-κB and improve MUC2, ZO-1 and occludin in DSS colitis (11). N5 can rebalance splenic Treg/Th17 responses and reduce NET-mediated gut-liver injury (13, 77). GLJ001 can shift the microbiota-SCFA-macrophage axis toward lower M1 polarization (60). Citrobacter infection studies connect *L. johnsonii* to ER stress relief and reduced inflammatory responses (98). These papers are persuasive at the level of mechanism. They are less persuasive as direct evidence for feed additives because DSS and Citrobacter models are not post-weaning diarrhea, and mouse colon physiology is not pig small-intestinal production biology.

Human evidence is uneven. La1/NCC533 reduced *H. pylori*-associated gastritis and inflammatory chemokines in mice and cell systems, but a multicenter randomized Crohn disease trial of LA1 after ileo-cecal resection did not reduce early recurrence (21, 22). The negative trial is useful because it prevents a selective reading of the field. More recent human-linked studies often use patient samples to show that *L. johnsonii* or its metabolites are reduced, then use rodents or cells for mechanism. IBD EV studies, CKD studies and immunotherapy-metabolite studies follow this pattern (57, 58, 65, 67). The human component adds relevance, but causality usually comes from animal or cell work. A mature review should state that difference directly.

Poultry and piglets are closer in production logic than mice and humans, yet their gut ecology differs. Poultry studies show that *L. johnsonii* can act as a competitive exclusion organism, but pathogen and strain specificity are clear (23, 70). Poultry host-specific genome studies also show that host-matched strains may colonize better or improve early performance (32). This supports the host-adaptation argument for piglets. It does not mean a poultry strain should be moved into pigs without testing. The same applies in reverse. Pig strains such as L531, RS-7 or N5/N7 may be more promising for swine because of source and existing data, but host source alone is not proof of efficacy.

Emerging niches expand the ecological range of the species. MT4 in oral mucosa shows that *L. johnsonii* can occupy a non-intestinal mucosal surface and attack *Candida* cell-wall integrity through chitinase activity (29). CRL1647 in bees shows a non-vertebrate association with colony-level benefits (30). Vaginal UBLJ01 studies show survival, adhesion and antimicrobial traits in a reproductive tract context (45). These findings should not be overextended. Their purpose in this review is to show that *L. johnsonii* is an adaptable host-associated species, with each niche imposing its own rules on persistence and function.

The cross-host pattern is uneven but useful. Piglets provide the strongest direct translational signal for post-weaning intestinal disease, especially through BNCC135265, RS-7 and L531. Rodents provide the deepest immune, barrier, metabolite and organelle mechanisms. Poultry provides clear colonization-resistance examples. Human studies provide caution, relevance and selected clinical anchors, including a negative trial. EV and postbiotic studies provide a new layer of effectors that may eventually be easier to standardize than live bacteria. The next stage of the field should connect these layers without pretending that a result in one host solves another host’s problem.

### Safety, manufacturing and standards for a strain-resolved field

Safety assessment for *L. johnsonii* has improved, but it remains uneven. IDCC 9203 underwent phenotypic and genomic safety evaluation, with no hemolysis, no beta-glucuronidase, no biogenic amines, low concern around tetW transfer and anti-inflammatory activity against LPS-stimulated macrophages (96). PF01 comparative genome analysis found no acquired resistance genes and interpreted many detected genes as survival, adhesion or BSH-related and not virulence factors (31). GJ231 work in beagles and mice included acid and bile tolerance, antimicrobial and antioxidant traits, CAZyme or bacteriocin potential and safety observations (44). These studies show the right direction, but a safety checklist should be attached to each candidate strain, not borrowed from a related strain.

Genome-based risk assessment should include acquired antimicrobial resistance, mobile genetic elements, plasmid context, virulence-factor searches, hemolysis, biogenic amine production, D-lactate risk where relevant, and stability during manufacturing. CRISPR and phage-defense content should be reported as evolutionary and production features, since phage susceptibility can affect fermentation consistency (33, 34). For livestock, compatibility with in-feed antibiotics, organic acids, zinc alternatives, enzymes and pelleting conditions must be tested. Many papers state CFU at dosing but not recovery after feed processing or storage. Without viability data at the point of consumption, efficacy cannot be reproduced.

Postbiotics and EVs create a different standard. A heat-killed product must define killing conditions, residual viability, active fractions, cell-wall integrity and batch consistency. An EV product must define vesicle isolation method, particle number, protein and lipid cargo, contaminating media components, endotoxin-like activity if any, storage stability and dose conversion across experiments. The N6.2 and colitis EV studies are mechanistically rich, but they also show how sensitive vesicle output can be to bile exposure, culture conditions and cargo composition (46, 54). A product cannot be described only as *L. johnsonii* EVs. It needs a preparation identity.

Clinical and livestock translation require negative controls that are more informative than vehicle alone. Comparisons with near-neighbor *Lactobacillus* species, heat-killed cells, supernatant, EV-depleted supernatant, purified effector and metabolite rescue can reveal whether a living strain is needed. Several studies already show the value of such comparisons. *L. johnsonii* outperformed *L. murinus* or *L. reuteri* in selected urate and calcium-metabolism models (62, 66). In PEDV-infected piglets, a different lactic acid bacterium and acetate were protective when *L. johnsonii* was not (68). In ALD, heat-killed *L. johnsonii* was highly active (16). These contrasts make the biology clearer and should become routine.

A final standard concerns language. Species-level abundance changes should be described as abundance changes. They should not be written as intervention evidence unless the organism is isolated and tested or depleted and rescued. Multistrain products should be described as multistrain products. If *L. johnsonii* is one component, attribution should be limited. Disease-specific efficacy should not be generalized to gut health without matching endpoints. This is not excessive caution. It protects the best *L. johnsonii* studies from being diluted by weaker claims.

### Research priorities for strain-resolved *L. johnsonii* biology

**Figure 2:**
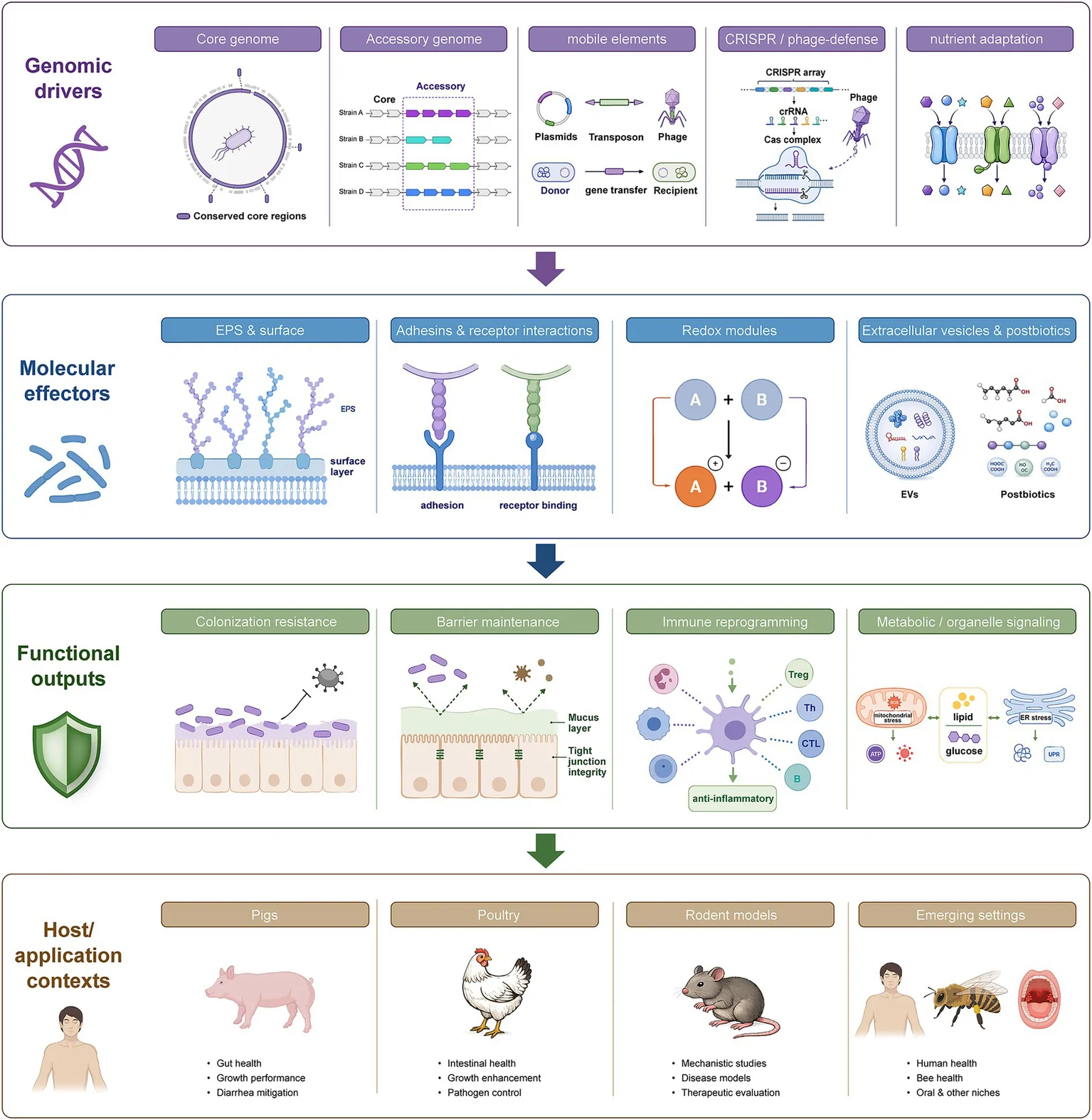
Mechanistic framework linking genomic variability in *L. johnsonii* to host phenotypes. Core and accessory genome features, host-adaptive traits and mobile genetic elements shape exopolysaccharide biology, adhesion-related proteins, redox behavior and non-cellular effectors such as extracellular vesicles and postbiotics. These properties converge on barrier, immune, metabolic and organelle-stress phenotypes that differ across host systems and disease contexts.

Future studies should move from static genome annotation to condition-specific strain function. The genomic-to-phenotype framework in Figure 2 provides a useful scaffold, but the next step is experimental linkage. Strain genomes should be paired with transcriptomes or proteomes under conditions that resemble the target niche, including bile exposure, low pH, oxygen gradients, feed carbohydrates, pathogen pressure and mucosal contact (99). Surface proteins, EPS loci, BSH genes, CRISPR and phage-defense systems, carbohydrate transporters, redox modules and secretion pathways should then be connected to measurable effectors rather than listed as genomic potential. Effector testing also needs to become more routine. EPS mutants, surface-protein blocking experiments, EV-depleted supernatants, purified vesicle cargo, heat-killed fractions and metabolite rescue can separate viable-cell effects from bacterial structures or metabolites. FI9785 EPS work, La1 GroEL studies, MG-GAPDH experiments, N6.2 EV and phospholipid studies, and HKLJ postbiotic work show that this level of resolution is feasible (16, 40, 41).

Host-context testing needs the same discipline. In pigs, prevention and treatment should be separated because pretreatment success does not guarantee rescue after diarrhea has begun. Trials should report sex, weaning age, diet, antibiotic background, zinc or acidifier use, pathogen exposure, baseline microbiota, dose stability and strain recovery. Multi-omics can be valuable, but only when linked to a mechanism question. Untargeted profiles without strain recovery, effector testing or functional rescue may generate attractive figures but weak attribution. Strain selection should also follow host ecology and evidence depth, as summarized in Figure 1 (100). Pig-source strains with direct or near-direct evidence, including L531, RS-7, BNCC135265 and N5/N7, are natural candidates for swine work. PF01 and ZLJ010 are useful genomic resources but still need modern efficacy testing in piglets (24, 25). N6.2 is better viewed as a model for redox, vesicle and phospholipid biology than as an immediate piglet candidate (15, 28). FI9785 remains a poultry colonization-resistance benchmark (23), and MT4 defines an oral mucosal anti-fungal niche (29). This allocation helps prevent strain shopping, where a favorable mechanism from one strain is used to justify another strain without comparable evidence.

Human translation should follow the same restraint. The negative LA1 Crohn trial should remain part of the evidence base rather than being treated as an exception (22). Human-linked and disease-model studies connect *L. johnsonii* to human disease biology through samples, metabolites and tissue signals (57, 58, 65, 67), but they still require controlled testing with defined strains or preparations. The same applies to heat-killed cells and EV products. Human studies would benefit from the practical discipline of livestock trials, where dose, stability, pathogen pressure, delivery format and fecal recovery are central variables rather than secondary details.

A useful benchmark is whether a study can name the strain, host, preparation, effector and phenotype in one testable statement. “*L. johnsonii* improves gut health” is not enough. The field would benefit from fewer species-level claims and more studies with clear strain identity, defined mechanisms, and endpoints linked to specific host contexts.

## Conclusion

*Lactobacillus johnsonii* is best understood as a host-associated species with strain-dependent functions, not as a uniform probiotic entity. In weaned piglets, the strongest evidence comes from named strains that reduce post-weaning diarrhea or pathogen burden and link these outcomes to barrier support, immune regulation, antioxidant responses, SCFA maintenance and epithelial stress control. Cross-host studies add mechanistic depth by identifying surface structures, EPS, adhesins, moonlighting proteins, redox systems, bile acid and indole metabolism, extracellular vesicles and postbiotic fractions as possible effectors. These mechanisms are valuable, but they should not be transferred across strains or hosts without direct testing.

The field now needs sharper attribution rather than broader claims. Future studies should identify the strain, define the host setting, specify the preparation, measure the relevant effector and connect these elements to a clear phenotype. Some *L. johnsonii* strains may become livestock probiotics, others may be better suited as postbiotic or extracellular vesicle platforms, and some may remain tools for studying mucosal, metabolic or organelle biology. Transparent reporting of neutral or negative findings will also be valuable, because such results can help define context-specific boundaries, improve strain selection and reduce publication bias when interpreted together with positive efficacy and mechanistic evidence.
